# Establishing the Psychometric Properties of the ICOAP Questionnaire through Intra-Articular Treatment of Osteoarthritic Pain: Implementation for the Greek Version

**DOI:** 10.1155/2016/6201802

**Published:** 2016-03-13

**Authors:** George E. Manolarakis, Nick Kontodimopoulos, Dimitra Sifaki-Pistolla, Dimitris Niakas

**Affiliations:** ^1^Faculty of Social Sciences, Hellenic Open University, Patras, Greece; ^2^Sitia's General Hospital, Sitia, Crete, Greece; ^3^Medical School, Department of Social Medicine, University of Crete, Heraklion, Crete, Greece

## Abstract

*Objectives*. In this prospective study, we intend to establish the psychometric properties of ICOAP for its use in studies involving the Hellenic population.* Methods*. SF-36 Health Survey was used as a standard against ICOAP scores from a sample of 89 patients (mean age: 71.07, 69 females) with hip and knee OA pain who underwent 2 treatment cycles of 4 intra-articular injections of sodium hyaluronate, separated by a 12-week medication-free time interval. Both questionnaires were filled twice with no missing data during follow-up.* Results*. ROC analysis accomplished ICOAP's criterion-related validation. Wilcoxon Signed-Rank Test and paired samples *t*-test endorsed ICOAP's responsiveness along with Effect Size values, standard response mean, and Relative Efficiency. Comparisons between the areas under curves (AUC) on ROC plots established external responsiveness. Cronbach's-alpha value favored ICOAP's internal consistency. This, along with intraclass correlation, results in both advocated reliability and content validity. Interitem discrimination was demonstrated by the ease of completion of ICOAP as well as the degree of familiarity with it. These findings inaugurated construct validity in collaboration with Spearman's and One-Way ANOVA results.* Conclusions*. ICOAP is a valid, reliable, and responsive QoL instrument and suitable for studies of osteoarthritic joint pain in the Greek setting.

## 1. Introduction

During recent decades, the ongoing increase in life expectancy has shifted the interest of health professionals towards new ways of disease management. In the case of osteoarthritis (OA), this interest addresses the most important need of every arthritic patient: to live a pain-free life at the lowest functional compromise.

Keeping in mind a variety of conservative and surgical treatment methods that are still in use, it becomes obvious that osteoarthritic pain comes into play as a major health-related quality of life (HRQoL) determinant that challenges any country's health system efficiency in terms of burden of disease.

Among developed HRQoL questionnaires focused on osteoarthritic pain, the Intermittent and Constant Osteoarthritis Pain (ICOAP) questionnaire for hip and knee osteoarthritis, a relatively new assessment tool, is the first to introduce the distinction of OA hip and knee pain in its two components: constant (ICOAP-CP) and intermittent (ICOAP-IP) pain. This distinction provides detailed information for each of these two kinds of pain separately as well as for total pain, thus forming a global view which differs from “pain on activity” as measured by all the preexisted questionnaires [1].

In this study, we attempt to establish the psychometric properties, namely, validity, reliability, and responsiveness, of the Greek version of the ICOAP questionnaire from a sample of OA patients following a specific treatment protocol applied for hip and knee OA by injecting intra-articular Sodium Hyaluronate (HYNa). The whole process was guided by the attributes and criteria set by Scientific Advisory Committee of the Medical Outcomes Trust [2].

## 2. Methods

### 2.1. Eligibility Criteria

Eligible participants were individuals diagnosed with single joint (hip or knee) OA-related pain lasting for 3 months or more and meeting the clinical and radiographic criteria established by the American College of Rheumatology [3], along with Kellgren and Lawrence radiographic OA classification [4]. They also underwent lab tests to rule out infection or rheumatic/metabolic disorders.

All participants were native Greek language speakers and they provided informed consent to participate.

Ethical approval for the study was granted by the hospital's ethics committee in accordance with the principles of the Declaration of Helsinki [5].

### 2.2. Sampling

This is an experimental study with pharmacological intervention without control group. 89 patients with chronic, hip or knee, osteoarthritic pain in a single joint regardless of bilateral OA existence were enrolled in the study between August 2011 and February 2012. A subsample (part of the original sample) of 25 people with the same severity unilateral knee OA was formed exclusively for the test-retest reliability needs.

Prior to the conduction of the study, 50 subjects filled the ICOAP questionnaires and maximum SD among age groups was calculated (*σ* = 1.085). For a study population of 1101 OA individuals (proportionally calculated) based on age-weighted OA prevalence in Greece [6], 12% (*N* = 133) were expected to demonstrate pain [7, 8]. With 5% sampling error and CI 95%, it was found that 51 was the appropriate sample size (*n*) for the study (*n* = *n*_0_/1 + *n*_0_/*N*, whereas *n*_0_ = *z* ^2^ *∗σ* ^2^/*d* ^2^, *z* = 1.96, and *d* = 0.125). Raosoft's (http://www.raosoft.com/samplesize.html) online software set the final sample size to 89 in order to satisfy a 20% response distribution (20% for every possible answer in each question).

### 2.3. Treatment Protocol

All patients followed an 18-week therapeutic scheme consisting of two treatment cycles (phases A and C, resp.). Each phase lasted 3 weeks. Between these two phases, there was a 12-week period (phase B) without treatment. Each treatment cycle consisted of 4 intra-articular injections of Sodium Hyaluronate (HYNa) administrated once weekly.

The beneficial clinical effect of HYNa is known for at least two decades [9]. This kind of treatment is recommended among a number of applicable nonsurgical therapies for OA by the European League Against Rheumatism (EULAR) [10] while OARSI highlights its significant efficacy despite the conflicting conclusions among several studies [11, 12]. In general, these guidelines demonstrate good structure and established criterion validity. Furthermore, they address degenerative and functional alterations in joint cartilage and are related to satisfactory outcome “discrimination” among the applied treatment modalities [13].

Patients completed both SF-36 and ICOAP questionnaires twice: first at the beginning (pretreatment) and then just after the last injection. Questionnaires were collected immediately after their completion. Investigators were blinded throughout the study with regard to both the identity of the participants and the answers given.

### 2.4. Research Tools

#### 2.4.1. ICOAP

The ICOAP questionnaire resulted from the collaboration between Osteoarthritis Research Society International (OARSI) and the Committee of Outcome Measures in Rheumatology (OMERACT). It is essentially a disease-specific research tool focused on osteoarthritic hip and knee joint pain.

ICOAP evaluates both constant pain and pain that “comes and goes.” It consists of 11 questions in its final form; the first five refer to constant pain and the remaining six to “intermittent” pain. Preliminary psychometric testing has shown the ICOAP to be reliable and valid [1] as well as responsive [14].

The main advantage of this assessment tool lies in the ascertainment that “intermittent pain” significantly impacts quality of life (QoL) especially when pain is intense and unpredictable. Among the 12 questions from the initial design of the instrument, one item, predictability of pain, was removed from subsequent analyses as correlations with other items and item-total correlations were low. This was attributed to its strong evidence of its subjectivity and the likelihood of degrading the questionnaire's psychometric properties [1].

Each question is scored from 0 to 4, and the sum of the 11 responses, as suggested by the developers, is further standardized on a scale between 0 (no pain) and 100 (worst outcome) [1].

Another important advantage that differentiates ICOAP from similar research tools is the fact that it raises questions about the distress and the effect of a painful state on a person's quality of life. These novelties have made it quite attractive for use [15].

ICOAP has been translated and cross-culturally adapted in parallel, using a common protocol, into 9 different languages other than English in order to test its adaptability to the specific cultural pattern of each society/nation, after which it became available for use in international multicenter studies [16]. Although it has also been available in Greek since 2007, it has not yet been validated for the Greek population.

#### 2.4.2. SF-36

The Health Survey SF-36 (Medical Outcomes Trust, Boston, MA) is a well-known multipurpose quality of life questionnaire having been used in numerous studies that measure the effects of various diseases [17].

It contains 36 questions which are followed by 2 to 6 possible answers. Each interviewee is asked to choose the response which best matches his/her actual health state.

Both structure and content are assigned to cover at least a minimum set of psychometric standards among those which are required for comparisons between different assessment groups.

The Medical Outcome Studies (MOS) team gave SF-36 its final form focusing on two main health aspects, the physical and the mental one, thus forming two concise indexes, the Physical Component Score (PCS) and the Mental Component Score (MCS), respectively. These 2 indexes were formed by 8 other preexisting scales that represent 8 different health components such as Physical Functioning (PF), Role Physical (RP), Bodily Pain (BP), General Health (GH), Vitality (VT), Social Functioning (SF), Role Emotional (RE), and Mental Health (MH). Ratings range between 0 and 100. Practically, the greater the score, the better the health status [18, 19].

The contribution of the Greek version of SF-36 research tool was essential for the study. Its translation procedure accommodated the guidelines of the International Quality of Life Assessment (IQOLA) Project followed by validation and reliability testing in the Greek population [18, 20, 21].

Although ICOAP seemed to be at first glance a disease-specific tool, it also showed quality of life clues as stated above. For that reason, we selected SF-36 as it can effectively measure both pain and QoL characteristics.

### 2.5. Statistics

IBM SPSS Statistics version 20 software was used for the statistical analysis. All continuous variables were assessed for normality of distributions with histograms and* Q*-*Q* plots and rechecked with the Shapiro-Wilk test.

Correlations were assessed either by Spearman's or Pearson's test according to the type of distribution of each variable. An excellent relationship was considered good if correlation coefficient was >0.9, fair if between 0.90 and 0.71, weak if between 0.70 and 0.51, little if between 0.50 and 0.31, and/or none if <0.3021.

Paired samples *t*-test along with One-Way ANOVA searched for differences between mean scores across treatment phases. The latter also investigated discriminant validity [22].

To test ICOAP's responsiveness, we used both Wilcoxon Signed-Rank Test and paired samples *t*-test. Estimations of the Effect Size (ES), Standardized Response Mean (SRM), and Relative Efficiency (RE) were considered to be essential in a study design like this whereas repeated measurements provide continuous data. In order to emphasize we took into account these indices along with Spearman's correlation coefficients. Effect Size was estimated by dividing the Mean Difference by the SD of baseline means, while in SRM the denominator was Mean Difference's SD. ES and SRM values lesser than or equal to 0.3 and equal to or greater than 0.8 disclosed low or large ES, respectively, while those within the intermediate interval exhibited moderate outcome [23].

ICOAP's Relative Efficiency was assessed by SRM ratios of ICOAP subscales over those of SF-36, while criterion-related validity was determined by ROC analysis which compared predictors of pain measured by ICOAP and SF-36's BP scales. The same analysis was carried out for SF-PF and VT scales. With the interest focused on the adequate combination of sensitivity and specificity, a dichotomous external outcome criterion (cut-off point) which best discriminates improved from unimproved conditions had to be defined [24]. While for some variables the “zero” (absence of pain) served as cut-off point, for others the median value (or its lowest 95% CI) means that all scores greater than this corresponded to positive outcome/improvement.

As the Area under Curve (AUC) in ROC plot depicts the magnitude of accuracy, AUC values of 0.5 (i.e., the area under the diagonal) or greater were considered to be of importance. The 95% CI lower limit served as criterion of statistical significance.

It is noteworthy that ROC analysis provides useful evidence of responsiveness along with paired samples *t*-test and Effect Size as proposed by Deyo et al. [25] (Table 3).

Convergent validation inquired high correlations between scales that measure the same/similar constructs, while divergent validity probed for scales that differ regarding the health aspect that they measure such as SF-VT and MH with those of ICOAP [26].

Based on ICOAP's Likert-pattern structure, its reliability was tested by Cronbach's-alpha coefficient [27] as well as test-retest reliability by intraclass correlation coefficient.

## 3. Results

Immediately after the export of descriptive statistics (Table 1), we proceeded to normality testing of variables which suggested the use of nonparametric statistical tests.

Statistically significant correlations were found in treatment's phase A between all ICOAP scales and SF-PF, SF-BP, and SF-PCS, with the same for SF-VT with ICOAP-CP (Constant Pain) and ICOAP-TP (Total Pain) scores. Regarding phase C, all ICOAP components showed strong relationships with all SF-36 scores, except SF-RE and SF-MH. Strong relationships were also detected between all ICOAP scores in both treatment phases (Table 2).

Paired samples *t*-test reported significantly improved mean scores in both ICOAP and SF-36 scores. Specifically, ICOAP showed improvement in CP, IP, and TP subscales at 50.27%, 45.71%, and 47.41%, respectively, while SF-36 disclosed similar results for PF (36.7%), RE (44.17%), RP (46.77%), and BP (67.57%) scores (Table 3).

Wilcoxon Signed-Rank Test affirmed statistically significant change in pain as implied by all ICOAP scores (*Z*_ICOAP  CP_ = −4.450, *Z*_ICOAP  IP_ = −5.797,  and  *Z*_ICOAP  TP_ = −6.042, *p* < 0.001) (Table 4).

One-Way ANOVA analysis revealed for phase A significantly high *F* values for both ICOAP subscales against SF-36 PF, BP, VT, and PCS scores (*p* value < 0.01) and moderate values for SF-VT (*p* value < 0.05) with ICOAP CP and IP demonstrating between 1.95 and 2.95 times higher *F* values as compared to SF-36 scales. The same was observed for phase C where *F* value was for ICOAP-CP 2,06 times and ICOAP-IP and 6.31 times higher, respectively, than each SF-36 scale (*p* value < 0.01) (Table 5)

Among scales with large ES values, SF-BP demonstrated the highest ES (1.27) followed by ICOAP-TP (0.88) and IP (0.84), as moderate ES ranked in descending sequence the following subscales: SF-PCS > SF-SF > SF-(PF &MH & MCS) > SF-VT > ICOAP-CP > SF-RE > SF-GH > SF-RP 23 (Table 3).

Relative Efficiency outcomes less than 1 were detected only in the BP scale and for ICOAP-CP with SF, MH, and PCS, whereas all the rest were >1, and in some cases >2 (e.g., RE with ICOAP-TP) (Table 6).

**Figure 1:**
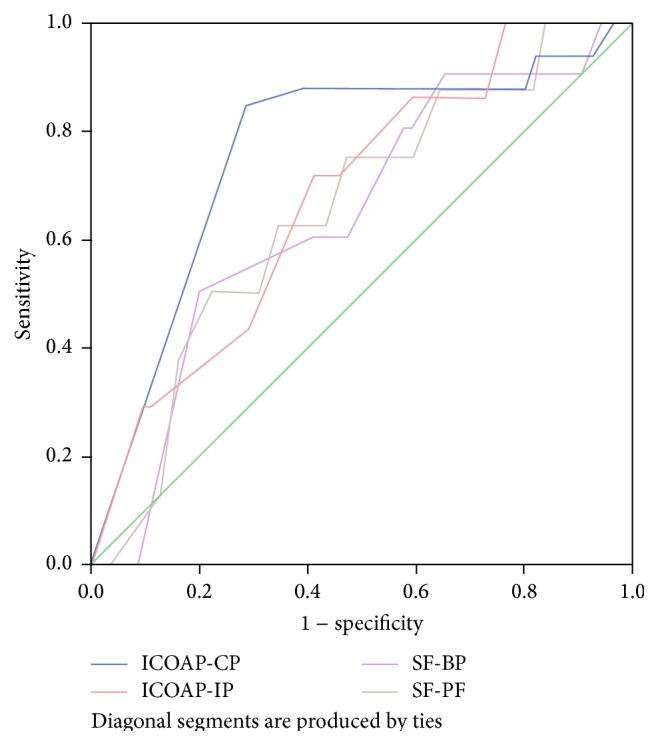
ROC curves.

By comparing the areas under curves (AUC) in ROC plots, ICOAP-CP demonstrated the best AUC while both SF-36 BP and PF demonstrated the worst AUC with ICOAP-IM to be laid in between them. Specifically, ICOAP-IP presented acceptable results while ICOAP-CP presented good results (according to the positive actual state that was used) against SF-BP and PF. It should be noted that we set the value 0.5 as the practical lower limit for each AUC (Table 7 and Figure 1).

Cronbach's-alpha coefficient showed in phase A excellent (>0.9) internal consistency for ICOAP CP and IP and almost excellent (0.878) internal consistency for ICOAP-TP, while showing excellent internal consistency in all scales for phase C (Table 8).

Test-retest reliability in a subsample of 25 patients with unilateral Kellgren-Lawrence III knee OA and during the same time frame showed excellent (>0.75) degree of approximation between them in terms of pain severity as registered by ICOAP scores (Table 8).

## 4. Discussion

This study attempts validation of ICOAP's Greek version through a specific conservative treatment intervention for osteoarthritis in patients with hip and/or knee joint OA pain and the use of SF-36 is deemed essential because it is a popular, well-established HRQoL research tool which meets reliability and validity criteria [28].

### 4.1. Face Validity

Face validity was established in a more empirical fashion as it was addressed on questionnaire's overall ease of use. Actually, ICOAP's interitem discrimination was established as easy as in SF-36. Participants clearly understood the purpose of each questionnaire. All questions were answered with the same degree of convenience while cases of completion inability were not reported due to lack of understanding.

### 4.2. Criterion-Related Validity

Criterion-related validity analysis explored evidence of the extent to which ICOAP scores are related to OA-pain, that is, its accuracy in “discovering” that kind of pain, as well as in quantifying it during the course of time, regardless of the intervention applied.

In an attempt to study the bilateral nature of a clinical outcome, one has to select the appropriate boundary. In ROC analysis, the definition of the proper cut-off level is of the highest importance because as that level decreases, sensitivity increases while specificity decreases and vice versa [29].

Besides the existence/absence of pain (zero cut-off point), we also took into account the assumption that a reliable questionnaire should capture changes to at least 50% of cases. For that reason, the lower limit of the 95% Confidence Interval was chosen as the alternative cut-off point.

Although power analysis showed that 52 was the minimum required sample size, bootstrapping to 1000 patients prior to ROC plotting upgraded the precision of results.

### 4.3. Construct Validity

Construct validation that was performed throughout the first treatment cycle explored the potential of ICOAP's overall construction to provide measurement results that warrant its task [30].

The absence of a control group halts construct validation according to Cronbach and Meehl, though ICOAP meets the criteria of the nomological network they have set [31].

As content and criterion-related validity along with interitem correlations are relevant to construct validity, the latter was demonstrated through these types of evidence as shown by associated tests and especially by high associations (Spearman correlation coefficients) or One-Way ANOVA results among the ICOAP components and SF-36 scales [32]. SF-BP seemed to be the most corresponding scale to ICOAP scores because it measures pain and its consequences in a person's daily activities. Anyway, Construct Validation requires numerous studies, not a single one [30].

Regarding Divergent Construct validation, we chose Pearson's c.c. for SF-VT against ICOAP TP because they both follow normal distributions.

### 4.4. Responsiveness

The magnitude of SRM values in accordance with that of ES concludes that ICOAP-IP and TP display higher responsiveness than ICOAP-CP, while SF-BP displays the highest one. Nevertheless, ICOAP's responsiveness was established by paired samples *t*-test results in conjunction with SRM, ES, and RE outcomes.

Values of RE > 1 in most scales provided evidence that the sample size was proper and capable for the detection of a specified ES [14].

It is meaningful that ES and SRM results were further confirmed by the highly significant “Wilcoxon Matched-Pair Signed-Rank Test” output. Practically, that high, in absolute values, ranked difference confirmed ICOAP's efficiency in reflecting posttreatment alterations in osteoarthritic pain (Table 3).

Attempting to confirm internal responsiveness, we estimated the magnitude of test-statistic value for each ICOAP subscale. Recorded values of *t*_0_ > 1.96 provided evidence of difference existence between two sequel measurements [33].

ROC analysis demonstrates an advantage over simple pre- and posttreatment comparisons in assessing scale responsiveness [27, 33]. Considering an AUC of at least 0.70 to be adequate, only ICOAP-CP and (almost) ICOAP-IP accomplished that task [34]. Obviously, ICOAP subscales seemed to be slightly better pain discriminators than those of SF-36 which had scores lower than that threshold [35] (Table 7).

This study showed almost the same results with preceding validations especially that of Bond et al. [23] which gave ESs and SRMs values within similar ranges. However, the type of treatment applied in each of these studies is of great significance as in treatment modalities with higher strength of recommendation (i.e., surgeries) one may expect better scores like those reported by Davis et al. [14].

#### 4.4.1. Ability of ICOAP to Respond to Changes

By estimating the depth of health outcome that ICOAP measured, we adopted the limit of 15% of patients with the lowest and highest scores, as proposed by McHorney and Tarlov [36]. With the data available, we noticed adequate effects: floor in ICOAP-CP, ceiling in SF-Social Functioning scale (phase C), and both ceiling and in SF-RP and RE. These findings for SF-36 match those from a previous study where responsiveness of WOMAC and SF-36 was tested in patients who had undergone hip replacement surgery [37].

ICOAP's floor effect was also reported in a study on patients with knee OA after treatment with physical therapy [22]. It is essential to underline that it is in line with SF ceiling effects because lower values in ICOAP correspond to better outcome (lesser pain) unlike in SF-36. So, the term “ceiling effect” of ICOAP seemed to be the most proper one instead of “floor effect.”

In an attempt to interpret these outcomes, one may hypothesize that extreme items may be missing in the lower ICOAP-CP scale with subsequent diminished content validity. The same hypothesis could be made for reliability as well as for responsiveness either because patients who scored at boundaries could not be further distinguished from one another or because changes between them could not be measured [34]. Such issues require a considerable number of studies in order to get a definite documented response.

Nevertheless, ICOAP shows comparable ability as compared to SF-36 in the detection of improvement after application of intra-articular treatment with HYNa.

### 4.5. Reliability

Since we explored ICOAP's ability to yield the same score on each administration to a given person and that score is of that person's true ICOAP outcome, we raised a reliability issue [38]. Cronbach's-alpha along with ICC coefficient both provided powerful results for achieving this. The “homogenous” subsample of 25 patients with unilateral K/L III knee OA served the test-retest reliability process which also reported excellent interrater agreement in respect to consistency/reproducibility of ICOAP measurements that were made by different observers.

#### 4.5.1. Internal Consistency

According to the “rule of the thumb” of George and Mallery, ICOAP demonstrated from the initial assessment excellent internal consistency for constant and intermittent pain (Cronbach's-alpha > 0.9) and almost excellent (0.878) internal consistency for Total Pain, while demonstrating excellent internal consistency for all scales at the end of treatment, revealing effective distinguishing of both types of osteoarthritic pain [39].

#### 4.5.2. Management of Measurement Error

This “experimental” study entails some degree of measurement error. Notwithstanding, the theory of reliability is based on the measurement of random error. Such a great proportion of common variance that is included in each item among paired observations (i.e., high Cronbach's-alpha values) means increased effectiveness in the management of measurement error [40].

Having already provided evidence of high construct validity, we can state that we have perhaps overcome the constant error issue. Indeed, ICOAP's structure contributed to versatile and well-rehearsed responses. Furthermore, the constant number of patients (no follow-up loss) throughout the study accomplished paired observations of the same parameter in the same sample. Lastly, high intraclass correlation coefficient outputs diminished the measurement error, as ICC estimates the average correlation among all possible orderings of pairs independently of the order of measurement [41].

It is generally admitted that larger correlation coefficients are associated with greater differences between measurement outputs (i.e., the initial and the final stage of treatment). This comes into agreement with the overall beneficial therapeutic outcome.

### 4.6. Content Validity

Construct validity subsumes all categories of validity [42] while content validation provides evidence about the construct validity of an assessment instrument [43]. Therefore, there's a reciprocal relationship between those two terms.

Cronbach's-alpha and ICC results provide extra evidence about the presence of Content Validity [44]. These outputs are in accordance with those that were reported for pain after knee replacement surgery [45].

### 4.7. ICOAP as HRQoL Instrument

During validation procedure, ICOAP revealed several QoL characteristics. Apart from the first 2 items of each subscale, all the rest provide QoL information on some aspects of patient's life that the pain potentially could affect, for example, individual's concerns or mood. Compared with SF-36, there are similarities among ICOAP's 4th and 10th questions and SF-36's 24th and 5th questions along with 11th and 26th questions, respectively. Any omission of a specific item barely affects the high Cronbach's-alpha value, providing evidence that each question contributes equally to the overall power of the questionnaire. Furthermore, the AUC results of ROC analysis demonstrated an adequate prediction level assuming that ICOAP provides additional information about OA pain-related quality of life [1, 44].

Despite the effort to identify some QoL characteristics of ICOAP, these are far from the original QoL character of SF-36 which still remains a benchmark for many researchers. This is confirmed by the associations made between ICOAP subscales and those of SF-36 which are not directly related to pain.

### 4.8. Limitations

During the study, we faced obstacles and dilemmas that should be reported. As posted previously, this study was not focused on a treatment's clinical effect, but on a questionnaire's validation. That is the reason that we did not form control group. Nevertheless, lack of control group prevents the strict application of Cronbach and Meehl's guidelines for construct validation and although the latter was achieved indirectly in any case, we recommend that these guidelines must be applied in any similar case.

Absence of a specific questionnaire for rating the significance of each ICOAP question can potentially exclude direct Content Validation; it is important to take this necessity into account for the future.

ICOAP-CP's “ceiling” effect could be attributed to the severity of OA in patients because 37% of knees and 64% of hips were rated minimal or mild OA (Kellgren/Lawrence I and II) where pain is not as intense as in advanced (K/L III and IV) stages. It is noteworthy that the therapeutic outcome was strongly influenced by HYNa's optimal effect as numerically shown by paired samples *t*-test mean scores or the percentage of patients who improved their ICOAP scores as well as by One-Way ANOVA *F* values.

However, neither Relative Efficiency nor the ceiling effect affected ICOAP's responsiveness which was further confirmed by other statistical tests as described above.

## 5. Conclusion

The ICOAP demonstrated strong agreement between the actual and the theoretically expected measurement of the constant and intermittent osteoarthritic hip/knee joint pain. Indeed, ICOAP can effectively introduce both sides of the same coin and it can also accurately quantify any possible variation in each pain subscale, displaying higher predictive ability than the most relevant (to pain) SF-36 scales.

As compared to SF-36, ICOAP shows comparable ability in detecting OA-pain and a discrete preponderance in recognizing any possible shift in its characteristics during interventions.

Based on the above-mentioned results, ICOAP fulfills its objective and displays a high comparability grade as well with other “similar” assessment tools. Its application for evaluation and management of both OA-pain types provides valid and comparable data.

Concluding, although ICOAP lacks standard QoL features, it is a valid, reliable, and responsive OA-pain instrument for use in studies relative to hip and knee osteoarthritis in the Greek clinical setting.

## Figures and Tables

**Table 1:** Descriptions.

Sample	Unilateral grade-3 knee OA subsample
Sample size: 89 pts (69 females–20 males)	Subsample size: 25 pts (18 females–7 males)
Mean age: 71.07 years (35–88)	Mean age: 72.88 years (67–80)
Mean BMI: 30.56 (39.3% overweight–47.2% obese)	BMI type: 13 overweight-12 obese
Knee pts: 77 - Hip pts: 12	Right/Left: 14/11

**Table 2:** Spearman's correlations.

	ICOAP constant pain (CP)	ICOAP intermittent pain (IP)	ICOAP total pain (TP)
	Corr. coef.	Corr. coef.
*Phase A*			
SF-36			
PF	−0.402^*∗∗∗*^	−0.390^*∗∗∗*^	−0.495^*∗∗∗*^
BP	−0.293^*∗∗*^	−0.534^*∗∗∗*^	−0.519^*∗∗∗*^
VT	−0.305^*∗∗*^	—	−0.346^*∗∗*^ (Pearson)
MH	−0.272^*∗∗*^	−0.417^*∗∗∗*^	−0.5^*∗∗∗*^
PCS	−0.350^*∗∗∗*^	−0.432^*∗∗∗*^	−0.494^*∗∗∗*^
ICOAP			
CP	—	—	0.738^*∗∗∗*^
IP	—	—	0.766^*∗∗∗*^
Age	—	—	0.255^*∗∗*^
*Phase C*			
SF-36			
PF	−0.413^*∗∗∗*^	−0.471^*∗∗∗*^	−0.610^*∗∗∗*^
RP	−0.330^*∗∗*^	−0.407^*∗∗∗*^	−0.523^*∗∗∗*^
BP	−0.457^*∗∗∗*^	−0.576^*∗∗∗*^	−0.696^*∗∗∗*^
GH	−0.312^*∗∗*^	−0.430^*∗∗∗*^	−0.527^*∗∗∗*^
VT	−0.394^*∗∗∗*^	−0.453^*∗∗∗*^	−0.564^*∗∗∗*^
SF	−0.461^*∗∗∗*^	−0.372^*∗∗∗*^	−0.556^*∗∗∗*^
RE	—	−0.430^*∗∗∗*^	−0.509^*∗∗∗*^
MH	—	−0.417^*∗∗∗*^	−0.500^*∗∗∗*^
PCS	−0.429^*∗∗∗*^	−0.487^*∗∗∗*^	−0.627^*∗∗∗*^
MCS	−0.293^*∗∗∗*^	−0.410^*∗∗*^	−0.497^*∗∗∗*^
ICOAP			
CP	—	0.287^*∗∗*^	0.690^*∗∗∗*^
IP	—	—	0.844^*∗∗∗*^

*p* value: ^*∗∗*^ *p* < 0.01, ^*∗∗∗*^ *p* < 0.001, and ^—^ *p* > 0.05.

**Table 3:** Paired samples *t*-test: ES and SRM.

	Means (SD)		Mean difference (SD)	Effect Size	Standardized Response Mean	Corr. coef.	Test statistic
	Phase A	Phase C	% diff.	(95% CI)	(95% CI)		
ICOAP							
Constant pain	29.60 (28.6)	14.72 (20.05)	14.88 (28.05) **−50.27**	0.52 (0.31–0.73)	0.53 (0.32–0.74)	0.377	5.007^*∗∗∗*^
Intermittent pain	41.99 (22.86)	22.80 (17.64)	19.19 (25.64) **−45.71**	0.84 (0.60–1.07)	0.75 (0.54–0.96)	0.219	7.062^*∗∗∗*^
Total pain	36.36 (19.48)	19.12 (15.43)	17.23 (21.32) **−47.41**	0.88 (0.65–1.11)	0.81 (0.6–1.02)	0.271	7.626^*∗∗∗*^
SF-36							
Physical Functioning	36.12 (23.66)	49.38 (26.55)	−13.26 (25.37) **+36.7**	−0.56 (−0.53)–(−0.57)	−0.52 (−0.73)–(−0.31)	0.494	−4.930^*∗∗∗*^
Role Physical	34.83 (42.41)	51.12 (44.26)	−16.29 (51.27) **+46.77**	−0.384 (−0.37)–(−0.371)	−0.32 (−0.53)–(−0.11)	0.301	−2.997^*∗∗∗*^
Bodily Pain	37.08 (19.69)	62.12 (21.20)	−25.04 (25.64) **+67.57**	−1.27 (−1.28)–(−1.25)	−0.97 (−1.18)–(−0.77)	0.215	−9.213^*∗∗∗*^
General Health	52.03 (20.81)	60.19 (19.85)	−8.16 (22.82) **+15.68**	−0.39 (−0.4)–(−0.38)	−0.36 (−0.56)–(−0.15)	0.371	−3.372^*∗∗∗*^
Vitality	56.29 (21.11)	67.64 (17.20)	−11.35 (21.63) **+20.16**	−0.54 (−0.58)–(−0.49)	−0.52 (−0.73)–(−0.31)	0.377	−4.949^*∗∗∗*^
Social Functioning	57.72 (25.39)	74.72 (24.49)	−16.99 (26.39) **+29.45**	−0.67 (−0.69)–(−0.647)	−0.64 (−0.85)–(−0.43)	0.440	−6.073^*∗∗∗*^
Role Emotional	44.94 (42.67)	64.79 (42.45)	−19.85 (49.93) **+44.17**	−0.46 (−0.47)–(−0.447)	−0.4 (−0.60)–(−0.19)	0.312	−3.751^*∗∗∗*^
Mental Health	62.74 (18.68)	73.17 (15.55)	−10.42 (19.29) **+16.6**	−0.56 (−0.6)–(−0.53)	−0.54 (−0.75)–(−0.33)	0.376	−5.099^*∗∗∗*^
Physical component	32.16 (8.59)	38.36 (10.34)	−6.2 (10.18) **+19.27**	−0.72 (−0.69)–(−0.76)	−0.61 (−0.82)–(−0.4)	0.434	−5.742^*∗∗∗*^
Mental component	46.61 (10.2)	52.33 (8.87)	−5.72 (11.39) **+12.27**	−0.56 (−0.59)–(−0.54)	−0.5 (−0.71)–(−0.29)	0.293	−4.735^*∗∗∗*^

*p* value: ^*∗∗∗*^ *p* < 0.001.

Bootstrap results are based on 1000 bootstrap samples.

**Table 4:** 

	Ranks				Wilcoxon Signed-Ranks Test	
	*N*	%	Mean rank	Sum of ranks	*Z*	*p* value
					Based on negative ranks	(two-tailed)
ICOAP constant pain (phase A versus phase C)						
Negative	12		24.67	296.00		
Positive	47	52.81	31.36	1474.00		
Ties	30					
					−4.450	0.000

ICOAP intermittent pain (phase A versus phase C)						
Negative	16		28.03	448.50		
Positive	66	74.16	44.77	2954.50		
Ties	7					
					−5.797	0.000

ICOAP total pain (phase A versus phase C)						
Negative	14		33.43	468.00		
Positive	72	80.89	45.46	3273.00		
Ties	3					
					−6.042	0.000

**Table 5:** One-Way ANOVA (by factor: ICOAP-TP).

Dependent variables	Sum of squares		Mean square		*F*
	Between groups	Within groups	Between groups	Within groups	
Phase A					
(Degrees of freedom: between groups, 30; within groups, 58; and total, 88)					
Physical Functioning	25542.224	23745.417	851.407	409.404	2.080^*∗∗∗*^
Bodily Pain	18244.283	15876.167	608.143	273.727	2.222^*∗∗∗*^
Vitality	18235.154	20991.250	607.838	361.918	1.679^*∗∗*^
Physical Health-PCS	3437.296	3066.246	114.577	52.866	2.167^*∗∗∗*^
ICOAP-CP	51554.986	20406.250	1718.500	351.832	4.884^*∗∗∗*^
ICOAP-IP	31843.740	14171.007	1061.458	244.328	4.344^*∗∗∗*^

Phase C					
(Degrees of freedom: between groups, 24; within groups, 64; and total, 88)					
Physical Functioning	34498.095	27542.917	1437.421	430.358	3.340^*∗∗∗*^
Role Physical	80898.057	91489.583	3370.752	1429.525	2.358^*∗∗∗*^
Bodily Pain	26873.786	12691.854	1119.741	198.310	5.646^*∗∗∗*^
Vitality	15240.744	10813.750	635.031	168.965	3.758^*∗∗∗*^
Social Functioning	28376.441	24429.036	1182.352	381.704	3.098^*∗∗∗*^
Role Emotional	76896.223	81680.556	3204.009	1276.259	2.510^*∗∗∗*^
Mental Health	9397.472	11889.000	391.561	185.766	2.108^*∗∗∗*^
Physical Health-PCS	6022.061	3394.435	250.919	53.038	4.731^*∗∗∗*^
Mental Health-MCS	3398.914	3521.719	141.621	55.027	2.574^*∗∗∗*^
General Health	19387.182	15298.571	807.799	239.040	3.379^*∗∗∗*^
ICOAP-CP	28784.957	6583.021	1199.373	102.860	11.660^*∗∗∗*^
ICOAP-IP	22827.412	4571.542	951.142	71.430	13.316^*∗∗∗*^

*p* value: ^*∗∗*^ *p* < 0.01, and ^*∗∗∗*^ *p* < 0.001.

**Table 6:** Relative efficiency.

SF-36	ICOAP		
	Constant pain	Intermittent pain	Total pain
Physical Functioning	1.02	1.44	1.56
Role Physical	1.65	2.34	2.53
Bodily Pain	0.54	0.77	0.83
General Health	1.47	2.08	2.25
Vitality	1.02	1.44	1.56
Social Functioning	0.83	1.17	1.26
Role Emotional	1.325	1.875	2.025
Mental Health	0.98	1.39	1.5
Physical Component Score	0.87	1.23	1.33
Mental Component Score	1.06	1.5	1.62

**Table 7:** ROC plots, Areas Under the Curves.

Scoring scale	Positive actual state	AUC	Std. error	*p* value	Asymptotic 95% CI	
					Lower bound	Upper bound
ICOAP-CP blue line	0	0.762	0.056	0.000	0.653	0.871
ICOAP-IP red line	37.50	0.684	0.096	0.108	0.496	0.872
SF-BP purple line	32	0.647	0.090	0.132	0.470	0.824
SF-PF brown line	35	0.653	0.095	0.156	0.467	0.838

Null hypothesis: true area equal to or greater than 0.5.

Bootstrap results are based on 1000 bootstrap samples.

**Table 8:** Reliability.

	Internal consistency		
	(Cronbach's-alpha coef.) (*N* = 89)		
ICOAP scales	Phase A	Phase C	Test-retest
			(Intraclass correlation coef.)
			On average measures (*N* = 25)
Constant pain (5 items)	0.959	0.956	0.910
	If item is deleted	
1	0.944	0.946
2	0.942	0.939
3	0.969	0.961
4	0.946	0.941
5	0.941	0.937
Intermittent pain (6 items)	0.914	0.923	0.877
	If item is deleted	
6	0.899	0.922
7	0.896	0.908
8	0.920	0.919
9	0.890	0.903
10	0.894	0.899
11	0.893	0.899
Total pain (11 items)	0.878	0.911	0.880

## Appendix

See Tables 2, 3, 4, 5, and 6.
